# Electromagnetic tracking system with reduced distortion using quadratic excitation

**DOI:** 10.1007/s11548-013-0925-4

**Published:** 2013-08-06

**Authors:** Tomasz Bien, Mengfei Li, Zein Salah, Georg Rose

**Affiliations:** Chair for Healthcare Telematics and Medical Engineering, Otto-von-Guericke University, Magdeburg, Germany

**Keywords:** Electromagnetic tracking, Computer-assisted surgery, Minimally invasive surgery, Compensation of conductive distortion

## Abstract

**Purpose:**

Electromagnetic tracking systems, frequently used in minimally invasive surgery, are affected by conductive distorters. The influence of conductive distorters on electromagnetic tracking system accuracy can be reduced through magnetic field modifications. This approach was developed and tested.

**Methods:**

The voltage induced directly by the emitting coil in the sensing coil without additional influence by the conductive distorter depends on the first derivative of the voltage on the emitting coil. The voltage which is induced indirectly by the emitting coil across the conductive distorter in the sensing coil, however, depends on the second derivative of the voltage on the emitting coil. The electromagnetic tracking system takes advantage of this difference by supplying the emitting coil with a quadratic excitation voltage. The method is adaptive relative to the amount of distortion cause by the conductive distorters. This approach is evaluated with an experimental setup of the electromagnetic tracking system.

**Results:**

In vitro testing showed that the maximal error decreased from 10.9 to 3.8 mm when the quadratic voltage was used to excite the emitting coil instead of the sinusoidal voltage. Furthermore, the root mean square error in the proximity of the aluminum disk used as a conductive distorter was reduced from 3.5 to 1.6 mm when the electromagnetic tracking system used the quadratic instead of sinusoidal excitation.

**Conclusions:**

Electromagnetic tracking with quadratic excitation is immune to the effects of a conductive distorter, especially compared with sinusoidal excitation of the emitting coil. Quadratic excitation of electromagnetic tracking for computer-assisted surgery is promising for clinical applications.

## Introduction

An electromagnetic tracking system is a navigation system that is based on the measurement of electromagnetic fields with a known distribution. Based on those measurements, the position and orientation of the field sensor can be calculated. Electromagnetic tracking systems are not dependent on line-of-sight or rigid connections like in optical and mechanical tracking systems. It is used in computer-assisted surgery to track the position of the instrument relative to the patient’s anatomy [1]. It has been used successfully for navigation in ear, nose and throat surgery (ENT) as well as in cranial and spine applications. The availability of microsensors has also allowed for the use of EMTS in tightly controlled cardiac mapping and pulmonary applications. EMTS has also shown its benefit in laparoscopic ultrasonography, needle aspiration biopsy, ultrasound freehand tracking, brachytherapy, ultrasound bone registration, and has also been introduced in endoscopic and bronchoscopic procedures [1–7].

EMTS is often applied in environments that typically contain metallic objects (conductive, para- or ferromagnetic) or electronic devices that may affect the magnetic reference fields. Ferromagnetic materials alter the shape of the reference fields and consequently distort both the direct current (DC) and the alternating current (AC) EMTS [8–10]. The eddy currents induced by conductive materials generate secondary AC magnetic fields, which distort the reference field pattern [8, 11–13]. The resulting fields are difficult to determine or characterize analytically [8, 14]. Stray fields produced by electronic devices [4, 14] or power lines [9] also distort the reference field. Internal electronic components of EMTS may also have an influence on the accuracy of position measurements [14]. Distortions originating from electronic devices and eddy currents additively overlay the reference field. Errors caused by nearby metallic objects are the most serious problem in clinical applications [14]. In a minimally invasive surgery operating table, LCD displays, light, metal studded walls, C-arms, operating microscopes and surgical instruments placed in proximity to the field generator affect the accuracy of EMTS [9, 14–16].

Distortions in EMTS could be eliminated by placing potential sources of distortions far away from the region of interest (ROI). This is typically not possible in clinical applications without changing the medical workflow. There are many methods presented in literature for the compensation of distortions caused by metal devices fixed in position and orientation. A survey of such methods of calibration has been published [17].

Calibration techniques, however, cannot compensate dynamic distortions originating from mobile objects such as surgical tools or movable imaging equipment. In such circumstances, measurements could be averaged or filtered (e.g., with a Kalman filter) [18]. Another method utilizing reverse tracking has been claimed, whereby a magnetic field source is integrated within the medical device, i.e., the catheter to be tracked contains the magnetic field source while a sensor coil array is distributed outside the patient body [19]. This approach is less sensitive to external distortions by conductive objects but suffers from very weak magnetic fields which could be generated within a small volume inside the patient’s body. Another approach is the shielding of at least one side of the tracking system [20]. Aside from strategies to avoid distortions, there are also approaches for the detection and correction of the errors caused by conductive objects.

The detection of field distortion can be accomplished using two field sensors attached rigidly to the surgical tool. The known distance between the sensors is compared with the measured value. If the deviations exceed a given tolerance, an error message may be presented [9].

The correction of metallic disturbances can be based on redundant measurements [21]. In this manner, another approach measured the position using two frequencies [22]. The measurement with lower frequency was interpreted as less distorted and was used to correct the measurement with higher frequency. In order to decrease its influence on the accuracy of the tracking system, the medical instrument in use could be made from laminated conductive materials [23] similar to transformer cores. In the patent [24], the authors model distortion with RL circuits and claim to correct the measured voltage with the calculated parameter of the conductive distorter. The author of the patent [25] proposes to represent the conductive distorter as lag or lead–lag networks. The excitation of the emitting coil with at least two frequencies leads to the calculation of the parameters of this lead–lag network. The influence of the conductive distorters is removed by adjusting the magnetic fields based on the estimated parameters.

In contrast, the method of distortion compensation presented in this paper does not require modeling of the parameter of the conductive distorter. In proposed approach, the emitting coil is supplied with a quadratic excitation, which therefore allows the voltage induced in the sensing coil by the emitting coil to be distinguished from the voltage induced by the conductive distorter. Only the undistorted voltage induced in the sensing coil directly by the emitting coil is used for the position and orientation calculation.

## Methods

### Equivalent circuit

**Fig. 1:**
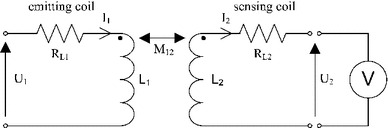
Equivalent circuit of the coupling between the emitting and the sensing coil

The presented method of the distortion minimization is based on a model of the tracking system and on a model of a conductive distorter. AC EMTS typically uses a sensing coil as a field sensor, which measures the magnetic field of the emitting coil for further position estimation. The equivalent circuit of the magnetically coupled emitting and sensing coil is presented in Fig. 1. In this equivalent circuit, the emitting coil is supplied with a voltage ${U}_{1}$ and the voltage ${U}_{2}$ is measured on the sensing coil. The amplitude of the voltage on the sensing coil is used for the position estimation. ${R}_{L1}, {R}_{L2}, {L}_{1}$ and ${L}_{2}$ are resistances and inductances of the emitting coil and of the sensing coil, respectively. The currents ${I}_{1}$ and ${I}_{2}$ are flowing in the emitting and in the sensing coil, respectively. $M_{12}$ is the mutual inductance between the emitting and the sensing coil.

The output of the model may be considered as an open loop due to the high impedance of the AD card. The current flow in the sensing coil ${I}_{2}$ is therefore approximately equal to zero. Such a system may be also considered as an air transformer with a very low efficiency.

The resulting transmittance ${G}_{1{-}2}(s)$ linking the input voltage ${U}_{1}$ and the output voltage ${U}_{2}$ is given in the Laplace domain in Eq. (1), where ${\tau }_{1}$ is a time constant depending on the inductance and on the resistance of the emitting coil (3).

1 $$\begin{aligned}&{G}_{1{-}2}({s})=\frac{{U}_2 ({s})}{{U}_1 ({s})}=\frac{M_{12}^{\prime } \cdot {s}}{\left( \frac{1}{{\tau }_{1}}+{s}\right) },\end{aligned}$$

2 $$\begin{aligned}&M_{12}^{\prime }=\frac{M_{12}}{{L}_{1}},\end{aligned}$$

3 $$\begin{aligned}&{\tau }_1 =\frac{{L}_{1}}{{R}_{L1}}, \end{aligned}$$

According to the transmittance, the voltage on the sensing coil is proportional to the derivative of the voltage on the emitting coil. The value of the mutual inductance $M_{12}$ is used for the calculation of the magnetic field of the emitting coil and for solving the position and orientation algorithm.

**Fig. 2:**
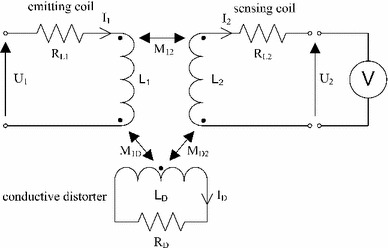
Equivalent circuit of two magnetically coupled coils with conductive distorter modeled as RL circuit

Conductive distorters in the EMTS may be modeled as an additional RL circuit [24, 25]. The equivalent circuit of two magnetically coupled coils with an additional conductive distorter is presented in Fig. 2. Along with the coefficients common with the previous model, Fig. 2 comprises the current flow within the conductive distorter ${I}_{\mathrm{D}}$, inductance ${L}_{\mathrm{D}}$ and resistance ${R}_{\mathrm{D}}$ of the conductive distorter. The model also includes the mutual inductance between the emitting coil and the distorter $M_{1{\mathrm{D}}}$ and the mutual inductance between the distorter and the sensing coil $M_{2{\mathrm{D}}}$.

The resulting transmittance ${G}_{1-D-2}(s)$ linking the input voltage ${U}_{1}$ and the output voltage ${U}_{2}$ of the two coils coupled indirectly across the RL circuit is presented in the Laplace domain in Eq. (4). The value of the time constant ${\tau }_\mathrm{D}$ of the conductive distorter (6) depends on the size, conductivity, form and the orientation of the conductive object relative to the magnetic field of the emitting coil. The voltage on the sensing coil is proportional to the second derivative of the voltage on the emitting coil.

4 $$\begin{aligned}&{G}_{1-D-2} ({s})=\frac{{U}_2 (s)}{{U}_1 (s)}=\frac{\frac{M_{1\mathrm{D}}}{{L}_1}\cdot {s}}{\left( {\frac{1}{{\tau }_1 }+{s}} \right) }\cdot \frac{\frac{M_{\mathrm{D}2}}{{L}_\mathrm{D}}\cdot {s}}{\left( {\frac{1}{{\tau }_\mathrm{D}}+{s}}\right) }\nonumber \\&\qquad \qquad \qquad \quad =\frac{M_{1\mathrm{D}2}^{\prime } \cdot {s}^{2}}{\left( {\frac{1}{{\tau }_1}+{s}} \right) \cdot \left( \frac{1}{{\tau }_{\mathrm{D}} +{s}} \right) },\end{aligned}$$

5 $$\begin{aligned}&M_{1\mathrm{D}2}^{\prime } =\frac{M_{1\mathrm{D}}}{{L}_{1}}\cdot \frac{M_{1\mathrm{D}}}{{L}_\mathrm{D} },\end{aligned}$$

6 $$\begin{aligned}&{\tau }_\mathrm{D} =\frac{{L}_\mathrm{D}}{{R}_\mathrm{D} }, \end{aligned}$$

The transmittance ${G}_\mathrm{E}(s)$ linking the input voltage ${U}_{1}$ and the output voltage ${U}_{2}$ including both the directly and indirectly induced voltages (7) is a sum of both transmittances (1) and (4).

7 $$\begin{aligned} {G}_\mathrm{E} (s)=\frac{{U}_2 (s)}{{U}_1 (s)}=\frac{M_{12}^{\prime } \cdot {s}}{\left( {\frac{1}{{\tau }_1}+{s}} \right) }+\frac{M_{1\mathrm{D}2}^{\prime } \cdot {s}^{2}}{\left( {\frac{1}{{\tau }_1 }+{s}} \right) \cdot \left( \frac{1}{{\tau }_\mathrm{D}}+{s} \right) }, \end{aligned}$$

The proposed model of the conductive distorter exhibits properties of a high-pass filter and therefore contributes additional filtered voltage to the voltage induced directly by the emitting coil. The transfer function is similar to the transfer function proposed by Schneider [25]. However, the model presented in this work links the voltage on the emitting coil with the voltage on the sensing coil. This difference is crucial for understanding the approach proposed in the following section.

The response of the system to a sinusoidal excitation without distortions is presented in the Laplace domain (8) and in the time domain (9), respectively.

8 $$\begin{aligned} {U}_{\mathrm{WS}} (s)&= {U}_1 (s)\cdot {G}(s)=\frac{{U}\cdot {\omega } }{\left( {{\varpi } ^{2}+{s}^{2}} \right) }\cdot \frac{{s}\cdot M_{12}^{\prime } }{\left( {\frac{1}{{\tau }_1 }+{s}} \right) }\nonumber \\&= \frac{{U}\cdot {\omega } \cdot {s}\cdot M_{12}^{\prime } }{\left( {{\varpi }^{2}+{s}^{2}} \right) \cdot \left( {\frac{1}{{\tau }_1 }+{s}} \right) },\end{aligned}$$

9 $$\begin{aligned} {U}_{\mathrm{WS}} ({t})&= {U}\cdot M_{12}^{\prime } \nonumber \\&\quad \cdot \frac{{\omega } \cdot {\tau } _{1}\cdot \left( {\cos \left( {{\varpi } {t}} \right) \!-\!\mathrm{e}^{-\frac{{t}}{{\tau }_1 }}\!+\!{\omega } \cdot {\tau }_1 \cdot \sin ({\varpi } {t})} \right) }{\left( {{\varpi }^{2}\cdot {\tau }_1^2 +1} \right) },\nonumber \\ \end{aligned}$$

The response may be distinguished between a steady-state response comprising a sum of sinusoidal functions and a transient response comprising an exponential function. After a time period depending on the time constant ${\tau }_{1}$, the transient response decays to zero and the response comprises only the sum of sinusoidal voltages.

The response of the system to a sinusoidal excitation caused by distortions only is presented in the Laplace domain (10) and in the time domain (11), respectively, where U is the amplitude of the sinusoidal excitation.

10 $$\begin{aligned} {U}_{\mathrm{DS}} (s)&= {U}_1 (s)\cdot {G}_{1{-}2} (s)=\frac{{U}\cdot {\omega }}{\left( {{\varpi }^{2}+{s}^{2}} \right) }\nonumber \\&\quad \cdot \frac{{s}^{2}\cdot M_{1\mathrm{D}2}^{\prime } }{\left( {\frac{1}{{\tau }_1}+{s}} \right) \cdot \left( {\frac{1}{{\tau }_\mathrm{D}}+{s}} \right) }\nonumber \\&= \frac{{U}\cdot {\omega } \cdot {s}^{2}\cdot M_{1\mathrm{D}2}^{\prime } }{\left( {{\varpi } ^{2}+s^{2}} \right) \cdot \left( {\frac{1}{{\tau }_1}+{s}} \right) \cdot \left( {\frac{1}{{\tau }_\mathrm{D}}+{s}}\right) }, \end{aligned}$$

11 $$\begin{aligned}&{U}_{\mathrm{DS}} ({t})={U}\cdot M_{1\mathrm{D}2}^{\prime } \cdot \frac{{\omega } \cdot {\tau }_1\cdot {\tau }_\mathrm{D} \cdot \mathrm{e}^{-\frac{{t}}{{\tau }_1}}}{\left( {{\varpi }^{2}\cdot {\tau } _1^2 \!+\!1}\right) \cdot \left( {{\tau }_1 \!-\!{\tau }_\mathrm{D}}\right) }\nonumber \\&\!\quad -{U}\cdot M_{1\mathrm{D}2}^{\prime } \cdot \frac{{\omega } \cdot {\tau }_{1} \cdot {\tau }_\mathrm{D} \cdot \mathrm{e}^{-\frac{{t}}{{\tau }_\mathrm{D}}}}{\left( {{\varpi } ^{2}\cdot {\tau }_\mathrm{D}^2 +1} \right) \cdot \left( {{\tau }_1 -{\tau }_\mathrm{D}} \right) }\nonumber \\&\!\quad + {U}\cdot M_{1\mathrm{D}2}^{\prime }\cdot \frac{{\omega }^{2}\cdot {\tau } _1 \cdot {\tau }_\mathrm{D} \cdot \left( {\omega } \cdot {\tau }_{1} \cdot \cos \left( {\varpi } {t} \right) -\sin ({\varpi } {t})\right) }{\left( {\varpi }^{2}\cdot {\tau }_{1}^{2} +1\right) \cdot \left( {\varpi }^{2}\cdot {\tau }_\mathrm{D}^2 +1\right) }\nonumber \\&\!\quad +{U} \cdot M_{1\mathrm{D}2}^{\prime } \cdot \frac{{\omega }^{2} \cdot {\tau }_{1} \cdot {\tau }_\mathrm{D} \cdot \left( {\omega } \cdot {\tau }_\mathrm{D} \cdot \cos \left( {\varpi } {t} \right) \!+\!\omega ^{2}\cdot {\tau }_{1} \cdot {\tau }_\mathrm{D} \cdot \sin \left( {\varpi } {t} \right) \right) }{\left( {\varpi }^{2}\cdot {\tau }_{1}^{2} \!+\!1\right) \cdot \left( {\varpi }^{2} \cdot {\tau }_\mathrm{D}^2 \!+\!1\right) },\nonumber \\ \end{aligned}$$

The sum of the voltage induced directly (9) and indirectly (11) in the sensing coil builds a resulting voltage. The voltage induced by the conductive distorter has the same frequency as the voltage induced directly by the emitting coil, and therefore, the distortion due to the conductive distorter cannot be filtered out of the measured sum of signals. The only feature which distinguishes the voltage induced in the sensing coil by the emitting coil from the voltage induced by the conductive distorter is a slightly different phase shift.

### Quadratic excitation

As a solution to the problem of distinguishing the voltage induced in the sensing coil directly by the emitting coil and indirectly by the conductive distorter, an EMTS with a quadratic excitation on the emitting coil (12) is proposed.

12 $$\begin{aligned} {U}_\mathrm{Q} ({t})={U}\cdot {t}^{2}, \end{aligned}$$

The response of the sensing coil on the quadratic excitation of the emitting coil without conductive distorter is presented in the Laplace domain (13) and in the time domain (14), respectively.

13 $$\begin{aligned} {U}_{\mathrm{WQ}} (s)&= X(s)\cdot {G}_{1{-}2} (s)=\frac{2\cdot {U}}{{s}^{3}}\cdot \frac{{s}\cdot M_{12}^{\prime } }{\left( {\frac{1}{{\tau } _{1}}+{s}}\right) }\nonumber \\&= \frac{2\cdot {U}\cdot {s}\cdot M_{12}^{\prime } }{{s}^{3}\cdot \left( {\frac{1}{{\tau }_1 }+{s}} \right) },\end{aligned}$$

14 $$\begin{aligned} {U}_{\mathrm{WQ}} ({t})&= 2\cdot {U}\cdot {\tau }_{1} \cdot M_{12}^{\prime } \cdot \left( {t}-{\tau }_{1} +{\tau }_{1} \cdot \mathrm{e}^{-\frac{{t}}{{\tau }_{1}}} \right) , \end{aligned}$$

The system response on the sensing coil to the quadratic excitation caused by the conductive distorter only is presented in the Laplace domain (15) and in the time domain (16), respectively.

15 $$\begin{aligned} {U}_{\mathrm{DQ}} (s)&= X(s)\cdot {G}_{1{-}2} (s)\nonumber \\&= \frac{2\cdot {U}}{{s}^{3}}\cdot \frac{{s}^{2}\cdot M_{\mathrm{1D2}}^{\prime } }{\left( {\frac{1}{{\tau }_{1}}+{s}} \right) \cdot \left( \frac{1}{{\tau }_\mathrm{D}}+{s} \right) }\nonumber \\&= \frac{2\cdot {U}\cdot {s}^{2}\cdot M_{\mathrm{1D2}}^{\prime } }{{s}^{3}\cdot \left( {\frac{1}{{\tau }_{1}}+{s}} \right) \cdot \left( {\frac{1}{{\tau }_\mathrm{D}}+{s}}\right) }, \end{aligned}$$

16 $$\begin{aligned}&{U}_{\mathrm{DQ}} ({t})=2\cdot {U}\cdot M_{\mathrm{1D2}}^{\prime }\nonumber \\&\quad \quad \cdot \left( \frac{1}{{\tau }_1 \cdot {\tau }_\mathrm{D}}\!+\!\frac{1}{{\tau }_1 \cdot \left( {{\tau }_{1} -{\tau }_\mathrm{D}}\right) }\cdot \mathrm{e}^{-\frac{{t}}{{\tau }_{1}}}-\frac{1}{{\tau }_{\mathrm{D}} \cdot \left( {\tau }_{1} -{\tau }_\mathrm{D} \right) }\cdot \mathrm{e}^{-\frac{{t}}{{\tau }_{\mathrm{D}}}} \right) ,\nonumber \\ \end{aligned}$$

The directly induced voltage (14) comprises a saw tooth, a rectangular pulse and an exponential transient response. The voltage induced indirectly (16) by the distorter comprises a rectangular pulse and an exponential transient response only. This difference is caused by a double derivative of the signal induced by the emitting coil induced in the sensing coil indirectly across a conductive distorter (4). According to the model, a saw tooth signal form of the voltage is induced in the conductive distorter. After the second derivative, the signal form is changed into a rectangular wave with an additional exponential transient response. The missing saw tooth signal form distinguishes the signal induced in the sensing coil directly by the emitting coil from the signal induced indirectly across a conductive distorter in the sensing coil. It also provides a measurement of the amplitude of the signal induced directly by the emitting coil while neglecting the voltage induced by the source of distortion. However, the measured signal also includes the exponential transient response, which overlays the steady-state response. After a time period dependent on the properties of the conductive distorter, the transient response decays to zero and the signal comprises the saw tooth and the rectangular pulse only. The measurement of the saw tooth wave allows calculation of the mutual inductance between the emitting and the sensing coil. This value of the mutual inductance allows estimation of the position of the sensor without an influence from the conductive distorter.

**Fig. 3:**
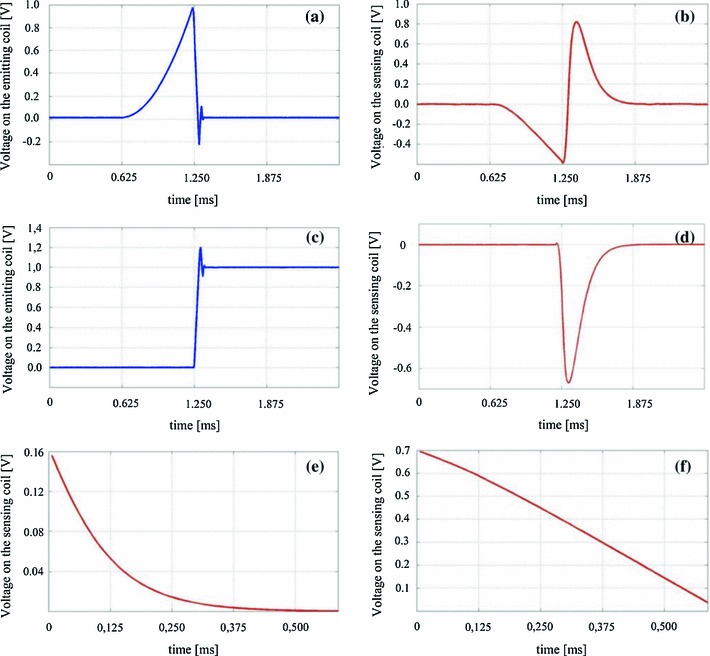
Extraction of the steady-state response from the response on the quadratic excitation of the emitting coil measured by ADC: **a** quadratic excitation fed into the emitting coil measured by ADC, **b** response on the sensing coil to the quadratic excitation of the emitting coil measured by ADC, **c** rectangular excitation fed into the emitting coil measured by ADC, **d** response on the sensing coil to the rectangular excitation of the emitting coil measured by ADC, **e** transient response of the system on the rectangular excitation estimated with double integration of the rectangular excitation of the emitting coil, **f** response on the sensing coil to the quadratic excitation with subtracted transient response

When the emitting coil is excited by quadratic excitation, the transient response in the form of the sum of exponential functions overlays the steady-state response comprising the saw tooth function. This problem could be solved by increasing the period of the quadratic excitation. However, the amplitude of the signal in the sensing coil depends on the time derivative of the current in the emitting coil. The increase in the length of the period would decrease the amplitude of the measured voltage in the sensing coil and therefore significantly reduce the working volume of the electromagnetic tracking system. In the presented paper, the transient response of the system is estimated in the first step using the response on the excitation by an additional rectangle pulse (Fig. 3c) supplied into the emitting coil shortly after supplying it with the quadratic excitation (Fig. 3a). The resulting response of the system (17) and of the conductive distorters (18) on a rectangle input voltage is as follows:

17 $$\begin{aligned} {U}_{\mathrm{WR}} ({t})&= {U}\cdot M_{\mathrm{\mathrm{1D2}}}^{\prime } \cdot \mathrm{e}^{-\frac{{t}}{{\tau }_{1}}},\end{aligned}$$

18 $$\begin{aligned} {U}_{\mathrm{DR}} ({t})&= {U}\cdot M_{\mathrm{1D2}}^{\prime } \nonumber \\&\quad \cdot \left( {\frac{{\tau }_{1}}{{\tau }_{1} -{\tau }_{\mathrm{D}}}\cdot \mathrm{e}^{-\frac{{t}}{{\tau }_1 }}-\frac{{\tau }_\mathrm{D} }{{\tau }_{1} -{\tau }_\mathrm{D}}\cdot \mathrm{e}^{-\frac{{t}}{{\tau }_\mathrm{D}}}} \right) , \end{aligned}$$

This response to a rectangular excitation contains information about the tracking system and the conductive distorter and comprises the transient response only (Fig. 3d). The emitting coil will be supplied with a rectangular signal directly after supplying it with the quadratic signal form. Therefore, it is assumed that the position of the sensing coil and the position of the conductive distorter relative to the emitting coils are constant over the entire position and orientation estimation. The response of the system to a rectangular input signal leads to the calculation of the transient response of the system to a quadratic input signal. This calculation is based on a relationship between the rectangular and the quadratic signal, that is, the second time derivative of the quadratic signal produces a rectangular form of the signal.

Therefore, the second numerical integration of the response on the rectangular wave with suitable amplitude is equal to the transient response of the system to the quadratic signal when the constant resulting from the numerical integration is removed. After the calculation, the estimation with the described method of the transient response of the system on the quadratic excitation (Fig. 3e) will be subtracted from the voltage measured on the sensing coil (Fig. 3f).

The transient response on the quadratic excitation is calculated with double integration of the response on the rectangle excitation:

19 $$\begin{aligned}&\int \int {{U}_{\mathrm{WR}} ({t})} \mathrm{dt}^{2}={U}\cdot M_{12}^{\prime } \cdot {\tau } _{1}^{2} \cdot \mathrm{e}^{-\frac{{t}}{{\tau }_{1}}},\end{aligned}$$

20 $$\begin{aligned}&\int \int {{U}_{\mathrm{DR}} ({t})} \mathrm{dt}^{2}={U}\cdot M_{\mathrm{1D2}}^{\prime } \nonumber \\&\quad \cdot \left( \frac{1}{{\tau }_{1} \cdot \left( {\tau }_{1} -{\tau }_{\mathrm{D}} \right) }\cdot \mathrm{e}^{-\frac{{t}}{{\tau }_{1}}}-\frac{1}{{\tau }_{\mathrm{D}} \cdot \left( {\tau }_{1} -{\tau } _\mathrm{D}\right) }\cdot \mathrm{e}^{-\frac{{t}}{{\tau }_\mathrm{D}}}\! \right) \!, \end{aligned}$$

The estimation of the position of the sensing coil requires calculation of the mutual inductance between every coupled emitting and sensing coil. The known dimensions of the emitting and sensing coil and the mutual inductance between the emitting and the sensing coil lead to the estimation of the position of the sensing coil. When the emitting coil is fed with the sinusoidal excitation, the mutual inductance is calculated based on the voltage measured in the sensing coil and the known amplitude of the voltage in the emitting coil. On the other hand, when the emitting coil is fed with the quadratic excitation, the mutual inductance between the emitting and the sensing coil will be calculated based on the gradient of the saw tooth signal induced in the sensing coil. Its curve is fitted with the least squares method. The gradient of the saw tooth signal leads to the calculation of the mutual inductance that consequently leads to the estimation of the position of the sensing coil.

## Experimental evaluation

### Experimental setup of the tracking system

**Fig. 4:**
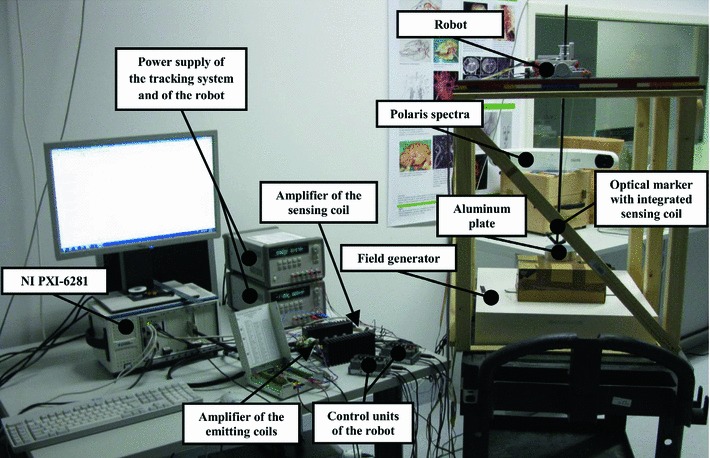
Experimental setup of the electromagnetic tracking with attached optical marker for the evaluation of the EMTS with quadratic excitation

For an experimental evaluation of the proposed method, an experimental setup of the tracking system was used (Fig. 4). It includes 8 emitting coils each with a diameter of 40 mm supplied with a voltage generated by an analog-to-digital converter NI PXI 6281 (ADC) and amplified by an amplifier. The experimental setup utilizes the NDI field sensor with a diameter of 0.3 mm as a sensing coil. The voltage induced in the sensing coil is amplified by an additional amplifier and measured by ADC. The resulting position of the sensing coil is estimated in Matlab by solving the position and orientation algorithm [26].

### Measurement setup

In multiple interventions in computer-assisted surgery, the sensing coil of the EMTS is attached to surgical instruments which are made of conductive materials. In the experimental validation of the proposed method, an aluminum disk with a radius of 0.05 m and a thickness of 0.03 m has been utilized as such a conductive distorter. This disk represents the surgical tools used in minimal invasive surgery, e.g., hammer, trocar, endoscope or ultrasonic probe. Those instruments are usually made of less electrically conductive and non-ferromagnetic steel. Therefore, in this experiment, the more electrically conductive aluminum disk may be considered as a worst-case scenario of distortion.

The goal of the performed experiment was to examine the influence of the conductive distorter on the accuracy of the tracking system with the quadratic excitation and compare it to the accuracy of the tracking system with the sinusoidal excitation. The sensing coil was moved within the working surface by a non-conductive plastic robotic arm in 200 random positions in proximity to the conductive distorter. The working surface had a rectangular shape ($0.1\hbox {m}\times 0.1\hbox {m}$), where the middle of the rectangle is placed in the middle of the coordinate system parallel to the surface of the field generator at a height of 0.15 m. The working surface was chosen to be relatively small because the experiment was designed in order to focus on the influence of the conductive distorter on the accuracy of the EMTS. The measurements were therefore performed in direct proximity to the conductive distorter in order to achieve the highest influence on the accuracy of the electromagnetic tracker. A larger distance between the sensing coil and emitting coils reduces the accuracy of the EMTS. Furthermore, the voltage on the sensing coil was measured for 8 s for the sinusoidal excitation (1 kHz) and 32 s for the quadratic excitation and averaged in order to increase the accuracy of the experimental setup of the tracking system.

After each movement of the robotic arm, the position of the sensing coil was measured by the electromagnetic tracking system and by the optical tracking system Polaris Spectra. Accuracy of this optical tracking system (root mean square error $<$0.25 mm) exceeds the expected accuracy of the EM tracking and therefore was used as a reference for the evaluation of the accuracy of the position estimation by the EMTS. The EMTS uses sinusoidal excitation in the first position measurement and shortly after, while in the same position, uses quadratic excitation. After 200 random measurements in the presence of the aluminum disk, the entire experiment was repeated without the presence of any conductive distorter.

In order to evaluate the electromagnetic tracking system, the coordinate system of the EMTS was registered with the coordinate system of the optical tracking system prior to the accuracy evaluation. For the performed registration, 27 additional measurements within a working volume of 0.2 m $\times$ 0.2 m $\times$ 0.2 m with both the electromagnetic and the optical tracking system were performed. The registration of both coordinate systems causes additional inaccuracy that increases the mean root square error of the position estimation with the optical tracking system. The registration error was calculated according to the work by Fitzpatrick [27]. The registration was carried out by fiducial registration via the software 3D Slicer.

## Results and discussion

### Results

**Fig. 5:**
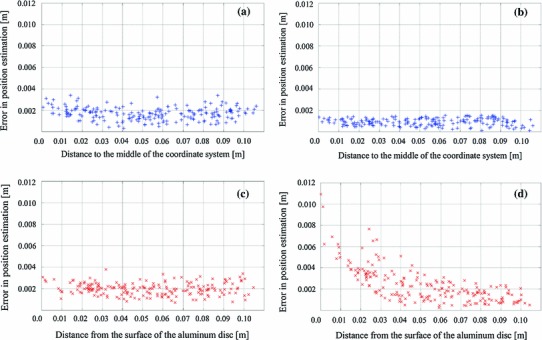
The influence of the aluminum disk on the accuracy of the electromagnetic tracking system as a function of the distance between aluminum disk and the sensing coil: **a** tracking with quadratic excitation without the aluminum disk, **b** tracking with sinusoidal excitation without the aluminum disk, **c** tracking with quadratic excitation nearby to the aluminum disk, **d** tracking with sinusoidal excitation nearby to the aluminum disk

The registration error introduced by registering the optical and electromagnetic tracking system is equal to 0.7 mm. The experimental errors in the position estimation described in the previous chapter are presented in Fig. 5. The errors were calculated in four different scenarios. Figure 5a, b presents the errors in the position estimation without the aluminum disk with the quadratic and sinusoidal excitation, respectively. Furthermore, Fig. 5c, d presents the errors in the position estimation with the aluminum disk with the quadratic and sinusoidal excitation, respectively.

The maximal error, the root mean square error (RMSE) and the standard deviation (std) for four different scenarios within the performed experiment are presented in Table 1.

**Table 1:** Accuracy of the electromagnetic tracker with and without the aluminum disk in the proximity

	Max error (mm)	RMSE (mm)	Std (mm)
Sinusoidal input voltage without conductive distorter	1.5	0.9	0.3
Quadratic input voltage without conductive distorter	3.4	1.7	0.6
Sinusoidal input voltage with conductive distorter	10.9	2.4	1.7
Quadratic input voltage with conductive distorter	3.8	2.0	0.6

### Discussion

According to Fig. 5, when the aluminum disk is placed in proximity of the sensing coil, the maximal error of the position estimation in the scenario with the sinusoidal excitation increased from 1.5 to 10.9 mm. The root mean square error of the position estimation increased from 0.9 to 2.4 mm. This shows a high influence of the conductive aluminum disk on the accuracy of the electromagnetic tracker. Despite the high increase in the maximal error of the position estimation in the sinusoidal excitation scenario with the conductive distorter in proximity to the sensing coil, the maximal error in the scenario with quadratic excitation changed only slightly from 3.4 to 3.8 mm. The RMSE with quadratic excitation changed from 1.7 mm without any distorters to 2.0 mm in the presence of conductive distorters. This increase in the RMSE in the presence of the distorter is smaller than the standard deviation of the measurements.

Additionally, Fig. 5c does not indicate the existence of a trend in the error measurement as the distance between the surface of the aluminum disk and the sensing coil increased for the quadratic excitation scenario. A declining trend could be seen, however, in Fig. 5d, where the sinusoidal excitation has been used. The results indicate very low influence of the conductive distorter on the accuracy of the electromagnetic tracker with quadratic excitation.

The root mean square error of the position estimation without the additional distorter for the EMTS with quadratic excitation (1.7 mm) is higher than with the sinusoidal excitation (0.9 mm). The lower accuracy in the position estimation is caused by the noise originating from, e.g., the amplifier, the discretization, the electromagnetic field generated by surrounding electronic devices or noise with a frequency of 50 Hz induced by the power supply. Due to the analysis of the system response on the quadratic excitation in the time domain, the response of the system was not filtered out. Only a low-pass filter of the ADC with a cutoff frequency equal to 80 kHz was used. In the proposed method, the emitting coils have been supplied with both the quadratic and rectangular signal form, which has a negative effect on the measuring rate.

## Conclusion

In this paper, a novel method of electromagnetic tracking is proposed, which by using quadratic excitation for position estimation reduces the error in the position estimation in the presence of the conductive distorter. The EMTS is able to compensate for dynamic distortions even when previous calibrations have not been made in the presence of the fixed distorting object. The proposed method is based on the difference in the transmittance between the voltage induced directly in the sensing coil and the voltage induced indirectly in the sensing coil across a conductive distorter. The undistorted voltage in the emitting coil depends on the first derivative of the voltage in the emitting coil. In contrast, the distorted voltage depends on its second derivative.

In many applications of computer-assisted surgery, the sensing coil is placed in direct proximity of conductive surgical instruments or even attached to such instruments. The proposed method was evaluated using an aluminum disk to represent those surgical instruments used in minimal invasive surgery like hammer, scalpel or ultrasonic probe.

In the performed experiment, the influence of the conductive distorter on the accuracy of the electromagnetic tracking system was measured simultaneously with a quadratic and a sinusoidal excitation. The measurements in the distance between the sensing coil and aluminum plate smaller than 20 mm show a non-significant impact of the conductive distorter on the accuracy of the electromagnetic tracking system when supplied with a quadratic excitation (Mann–Whitney $U$-test with the significance level 0.01). The same experiments performed with the sinusoidal excitation show a significant influence of the conductive distorter on its accuracy. On the other hand, it should be noted that the position estimation with the quadratic excitation is less accurate without additional distortions due to the conductive objects.

The methods proposed in the literature require parameter calculations of the conductive distorter equivalent circuit. These calculations are complicated and lead to high inaccuracies, especially when there are multiple distorters to be modeled as multiple RL circuits. In the proposed method, it is not necessary to calculate these parameters of the conductive distorter equivalent circuit, and therefore, the proposed method is applicable when multiple distorters with complicated geometry are present—a common situation in surgical and radiological suites.

The proposed method focuses on the distortions due to the conductive distortions and does not take into consideration distortions due to ferromagnetic distorters.
